# Genetic Polymorphisms and Cytokine Profile of Different Ethnicities inSeptic Shock Patients and their Association with Mortality

**DOI:** 10.5005/jp-journals-10071-23136

**Published:** 2019-03

**Authors:** Shahla Siddiqui, Resham Lal Gurung, Sylvia Liu, Edwin Chuen Ping Seet, Su Chi Lim

**Affiliations:** 1,4 Department of Anesthesia and Intensive Care, Khoo Teck Puat Hospital, Singapore; 2,5 Clinical Research Unit, Khoo Teck Puat Hospital, Singapore; 3 Diabetes Centre, Khoo Teck Puat Hospital, Singapore.

**Keywords:** Cytokines, Genomics, Radial profiles, Sepsis, Septic shock

## Abstract

**Objective:**

The outcomes of sepsis and septic shock patients are heterogonous, with avariable response despite standardized care. The aim of this study was toexplore the racial differences in septic shock outcomes, and theirassociation with genetic polymorphisms and cytokine levels in an Asianpopulation.

**Materials and methods:**

This was an observational cohort study with Intensive Care units of a 500bedded tertiary care hospital in Singapore. 198 patients (73 Chinese, 73Malay and 52 Indian and others) admitted to the Khoo Teck Puat HospitalIntensive Care Unit between August 2016 and June 2017, with a diagnosis ofsevere sepsis (according to) were enrolled. Plasma interlukin-6 (IL-6),interlukin-10 (IL-10) and tumour necrosis factor-a (TNFa) were measuredusing a highly sensitive quantitative sandwich enzyme-linked immunosorbentassay (ELISA) (BioVendor, Modrice, Czech Republic). The gene panel studiedincluded 16 genes.

**Results:**

The rs7038903 common variant in SVEP1 gene showed significant associationwith sepsis severity independent of other variants in ordinal logistic andlinear regression model (*p* = 0.001 and *p* = 0.002 respectively). Moreover, the association between rs7038903 and increased hazard for death remained significant after further adjusting for cytokines level. Interestingly, significant differences were seen in plasma IL6 inindividuals with or without rs7038903 C allele (28pg/ml (IQR 12-86) vs90pg/ml (IQR 49-155); P=0.022) in patients with severe sepsis in the Malayethnic group.

**Conclusion:**

Our study shows a promising polymorphism in SVEP1 gene (rs7038903) which isassociated with sepsis shock and 28 days mortality, independent of age, gender, and method of diagnosis and SOFA score. Collectively, while our findings so far have shown the additional value or measuring cytokines andgenetic markers in sepsis outcomes in the local population, further largescare studies are needed in a heterogeneous septic population with arigorous analysis to know the significance of our findings.

**How to cite this article:**

Siddiqui S, Gurung RL *et al.* Genetic Polymorphisms and Cytokine Profile of Different Ethnicities in Septic Shock Patients, and their Association with Mortality. Indian J Crit Care Med 2019;29(3):135-138.

## INTRODUCTION

Sepsis and septic shock remain leading causes of death globally, with a mortality rate close to 50%^1^. Sepsis is a multifactorial syndrome with a complex pro-inflammatory response leading to high morbidity and mortality^2^. The outcomes of sepsis and septic shock patients are heterogonous with a variable response despite standardized care^3^. Given the same treatment, some patients will recover and do well, whilst others will have poor outcomes. Several genes have been implicated after researchers turned their attention towards a more precise involvement of genetic variation^4^. Age, gender and ethnicity have been a focus of many studies in sepsis, but there is a large discrepancy in the results^5^. Racial variation in septic shock and mortality has been well documented. However, less is known about racial disparities among patients with sepsis and septic shock in a multi-ethnic Asian population. Singapore is composed of a predominantly Chinese population with a mix of Malay and Indians as well as foreigners. This ethnic mix produces an interesting cohort for such a study.

Cytokine and genomic studies can lead to early recognition and treatment of sepsis, as well as risk stratification^6^. Previous studies have inconsistently shown associations between genes and outcomes of sepsis^7^. Precision medicine studies have been changing practice for many diseases, and for an unpredictable and varied entity such as sepsis, it promises to be useful in the future^8^.

The aim of this study was to explore the racial differences in septic shock outcomes, and their association with genetic polymorphisms and cytokine levels in an Asian population.

## MATERIALS AND METHODS

### Study Population

In this observational cohort study, 198 patients (73 Chinese, 73 Malay and 52 Indian and others) admitted to the Khoo Teck Puat Hospital Intensive Care Unit between August 2016 and June 2017, with a diagnosis of severe sepsis (according to) were enrolled. The subjects belonged primarily to the three major Asian ethnic groups of Singapore. Q Sofa score and SIRS score was used for inclusion criteria. Absence of bacteremia was an exclusion criterion. The primary outcome was ‘septic shock’, defined as “profound circulatory, cellular, and metabolic abnormalities, associated with vasopressor requirement to maintain a mean arterial pressure of 65 mm Hg or greater and serum lactate level greater than 2 mmol/L (>18 mg/dL) in the absence of hypovolemia”^9^. The secondary outcome was all-cause 28-day mortality.

The study was approved by the Singapore National Health Group Domain Specific Review Board and written consent was obtained from each participant or their legally acceptable representative.

### Cytokine Measurement

Plasma interlukin-6 (IL-6), interlukin-10 (IL-10) and Tumour necrosis factor-a (TNFa) were measured using a highly sensitive quantitative sandwich enzyme-linked immunosorbent assay (ELISA) (BioVendor, Modrice, Czech Republic).

### Next-generation (Candidate Gene) Sequencing

DNA was extracted from the buffy coat of blood samples using a QIAamp DNA maxi kit (Qiagen, Mississauga, ON, Canada). The gene panel studied included 16 genes; IL10, PCSK9, AMPD1, FCGR2A, HSP, MBP, LDLR, IL1, IL1R, IL1RN, HIF-1, CD14, TNF-alpha, IL6, TLR4, and SVEP1 (Table 1). Specific primers were designed using Ion AmpliSeq Designer (Life Technologies, USA), covering all exons and 100 base pair of exon intron junction. The exome library was prepared using the Ion AmpliSeq Library Kit (Thermo Fisher Scientific, USA) and sequenced using Ion PGM Hi-Q Sequencing Kit and ‘318’ sequencing chips on Ion Torrent PGM System (Thermo Fisher Scientific, USA). Analyses were performed using Torrent Suite and Ion Reporter (Thermo Fisher Scientific, USA). Sequence reads were aligned to the human GRC37/hg19.

### Variant Calling

Variants were filtered to exclude coverage depth of <100 reads, synonymous variants. Only non-synonymous variant were selected for subsequent analysis. Functional prediction of variants were assessed with mutation prediction tools, including PolyPhen2 [17], SIFT [18], and MutationTaster2 [19], which are within the Alamut software. Only non-synonymous variant predicted to be functionally deleterious by at least two bioinformatics tools were selected for subsequent analysis.

### Statistical Analysis

Continuous variables were expressed as median and interquartile ranges (IQR). Categorical data were expressed as proportions. Participants were stratified by ethnicity and outcomes (septic shock and 28 days mortality status) to visualize clinical and biochemical characteristics. We used the Kruskal-Wallis test to test for differences in baseline characteristics for continuous data and x2 test for categorical variables. The association between cytokine levels and sepsis shock were evaluated using logistic regression model in all samples and separately by ethnic group. Non-normally distributed data were natural log-transformed to obtain normal distributions. Model was adjusted for demographic variables such as age, gender, surgical method and Qsofa. The association between cytokine levels and 28-days mortality were evaluated using Cox regression analysis adjusting for baseline age, gender, surgical method and Qsofa.

For association of genotype with septic outcomes, we first performed regression analysis (additive model) for association of 53 SNPs identified with septic shock and 28-day mortality. Given our small sample size in the study, we also defined sepsis severity outcome as severe sepsis, septic shock and uses of vasopressin and mortality within 28 days and performed 1) ordinal logistic regression analysis and 2) linear regression analysis. SNP with suggestive evidence of association with either of three outcomes were selected for subsequent multivariate association analyses. The Cox regression was used to test for differences in the hazard of death over 28 days according to genotype, adjusting for age, gender, a surgical versus a medical primary diagnosis, ethnicity, and qSOFA score. *p* value less than 0.05 were considered statistically significant. All analyses were performed using SPSS version 22 (SPSS Inc, IBM Corporation, Chicago, IL) and STAT/ SE 14.0 (StataCorp, College Station, Texas).

## RESULTS

Table 1 displays the baseline profile of the participants by ethnicity. Malay patients (median age 54.0, IQR 45-63) admitted were on average 9 years and 7 years younger than Chinese and Indian patients, respectively (*p* = 0.033) and the proportion of younger patients (< 65 years) were significantly higher (*p* = 0.006) in Malay (18%) as compared to Indian (28%) and Chinese (43%) (Table 1). BMI and proportion of obese patients were also higher in Malay compared to Chinese and Indian (*p* <0.001). The incidence of septic shock was comparable across ethnic groups (68.5% in Chinese, 61.1% in Malay and 61.5% in Indian,*p* = 0.421). However, relative to non-septic shock patients, significant increase in both pro-inflammatory cytokines IL-6 and TNFa in septic shock patients was observed only in Malay (*p* = 0.008, *p* = 0.002) (Table 2).

### Association of Cytokines and Septic Outcomes

Binary logistic regression analysis stratified by ethnicity and adjusted for age and gender revealed greater risk of septic shock associated with increased cytokines levels in Malay compared to Chinese or Indian. The 28 days all-cause mortality rate was between 10 to 12% in three different ethnic group. Malay patients with increased IL-6 and IL-10 levels had elevated risk for all-cause mortality in the cox regression analysis (hazard ratio (HR) = 2.45, 95% CI 0.95-6.36, p=0.065 for natural log transformed IL6 and HR =3.26, 95%CI 1.19-8.95, p=0.022 for natural log transformed IL10) (Table 3).

### Genetic Profiling

In our study, we focused on non-synonymous single-nucleotide polymorphisms (SNPs) that disrupt conserved regions of the genome and are predicted to have significant effects on protein function based on in silico bioinformatics tools. Functional prediction was assessed by four different bioinformatics algorithm* (Mutation Taster, GVGD, SIFT and PolyPhen-2). Only variants predicted to have deleterious effect on the function of protein by 2 or more tools were selected. Accordingly, we had identified 53 variants, of which 4 were common variants (Minor allele frequency (MAF) >5%), 2 variants with 5%>MAF >1% and remaining extremely rare. To identify variants potentially associated with sepsis outcomes, we undertook several strategy given our modest sample size in this pilot study. We examined the association of variants with sepsis severity (severe sepsis, septic shock and mortality) in ordinal logistic and linear regression model and identify specific SNPs with suggestive association. The rs7038903 common variant in SVEP1 gene showed significant association with sepsis severity independent of other variants in ordinal logistic and linear regression model (*p* = 0.001 and *p* = 0.002, respectively). According to Exome Aggregation Consortium (ExAC), minor allele frequency of rs7038903 is reported to be approximately 8% and 14 % in East Asian and South Asian respectively. We observed similar MAF for rs7038903 (6-8%) in our study population.

**Table 1:** Baseline profile of subjects stratified by ethnicity

*Characteristics*	*Chinese (n=73)*	*Malay (n=73)*	*Indian (n=52)*	*p-value*
Age (years)	63.0 (49.5-73.5)	54.0 (45.0-64.0)	61.0 (40.0-68.8)	**0.044**
> 65 years (n, %)	31 (42.5)	14 (19.2)	17 (27.9)	**0.010**
Female (n, %)	29 (39.7)	28 (38.4)	21 (27.3)	0.972
BMI (kg/m^2^)	22.1 (19.4-22.3)	27.1 (23.3-32.0)	22.6 (20.5-27.5)	**<0.001**
Obese (BMI>27.5kg/m^2^) (%)	12.7	47.0	24.4	**<0.0001**
SBP (mmHg)	123 (110-138)	126 (103-139)	119 (106-134)	0.601
DBP (mmHg)	69 (61-78)	70 (59-82)	67 (57-79)	0.594
qSOFA	2.0 (2.0-2.5)	2.0 (2.0-3.0)	2.0 (2.0-2.0)	0.296
*Cytokines*				
IL6 (pg/ml)	51.8 (13.1-198.7)	43.2 (13.9-98.5)	29.4 (13.9-59.9)	0.261
IL10 (pg/ml)	13.3 (7.0-28.8)	12.8 (7.7-25.7)	12.0 (6.5-23.6)	0.744
TNF-a(pg/ml)	7.7 (3.5-17.3)	7.4 (3.6-14.0)	5.8 (3.8-14.2)	0.873
*Outcome (n, %)*				
Sepsis shock	50 (68.5)	44 (60.3)	30 (57.7)	0.409
28 days Mortality	9 (12.3)	8 (11.1)	5 (9.8)	0.896
Length of Stay (days)	5.5 (2.0-10.0)	4.0 (3.0-8.5)	5.0 (3.0-8.8)	0.907

Data are presented as frequency (%) for categorical variables or median (25th-75th percentile).

**Abbreviations**: BMI, body mass index; SBP, systolic blood pressure; DBP, diastolic blood pressure; IL6, interlukin-6; IL10, interlukin-10; TNF-a, tumour necrosis factor alpha.

**Table 2:** Association of cytokine levels with development of septic shock and 28 days mortality stratified by ethnicity

	*Chinese*		*Malay*			*Indians*		*All samples*	
**Outcomes**	*Variable OR (95%CI)*		*p value OR (95%CI)*		*p value*	*OR (95%CI)*	*p value*	*OR (95%CI)*	*p value*
**Septic shock**	IL-6^‡^	**1.65 (1.12-2.42)**	**0.011**	**1.82 (1.20-2.76)**	**0.005**	1.67 (0.94-2.97)	0.083	**1.60 (1.27-2.02)**	**<0.0001**
	IL-10^‡^	**2.59 (1.40-4.81)**	**0.003**	**2.33 (1.16-4.69)**	**0.018**	1.97 (0.94-4.11)	0.072	**2.18 (1.50-3.17)**	**<0.0001**
	TNFa^‡^	**2.01 (1.23-3.28)**	**0.005**	**2.45 (1.36-4.42)**	**0.003**	1.47 (0.98-3.19)	0.327	**1.96 (1.43-2.68)**	**<0.0001**
**28 days mortality**		HR (95%CI)	*P value* HR (95%CI)		*P value*	HR (95%CI)	*P value*	HR (95%CI)	*P value*
	IL-6^‡^	1.12 (0.67-1.87)	0.659	**2.13 (1.16-9.93)**	**0.015**	3.12 (0.78-12.5)	0.107	**1.46 (1.11-1.92)**	**0.008**
	IL-10^‡^	0.99 (0.46-2.12)	0.977	**3.19(1.31-7.72)**	**0.010**	1.60 (0.66-3.87)	0.300	**1.53 (1.08-2.17)**	**0.016**
	TNFa^‡^	0.681(0.34-1.37)	0.283	**3.22(1.33-7.78)**	**0.009**	2.56 (0.44-15.06) 0.299		**1.62(1.01-2.60)**	**0.046**

Septic shock and 28 days mortality was measured by logistic regression and cox regression respectively adjusted for age, gender, surgical method, Qsofa and ethnicity (in all samples). ‡Natural log transformed. P<0.05 was presented in bold.

**Table 3:** Association of SNP with development of septic shock and 28 days mortality

		*Septic shock*		*Mortality*	
*Variable*	*OR (95% CI)*	*p value*	*HR (95% CI)*	*p value*
Unadjusted	rs7038903	2.44 (0.96-6.21)	0.060	2.14 (0.91-5.02)	0.080
Adjusted	Age	1.02 (1.00-1.04)	0.034	1.00 (0.97-1.02)	0.796
	Female vs male	0.69 (0.37-1.28)	0.241	1.08 (0.44-2.64)	0.866
	Indian vs Chinese	0.66 (0.30-1.43)	0.289	0.75 (0.25-2.27)	0.604
	Malay vs Chinese	0.68 (0.33-1.41)	0.303	0.74 (0.28-1.94)	0.548
	Medical vs surgical	1.00 (0.54-1.86)	0.994	1.35 (0.52-3.48)	0.540
	qSOFA	**1.63 (1.05-2.53)**	**0.030**	**5.10 (2.23-11.68)**	**<0.001**
	rs7038903	**2.58 (0.98-6.81)**	**0.056**	**2.96 (1.10-7.94)**	**0.031**

Septic shock and 28 days mortality was measured by logistic regression and cox regression respectively adjusted for age, gender, surgical method, Qsofa and ethnicity (in all samples). P<0.05 was presented in bold.

### Association of rs7038903 and Septic Outcomes

We next tested for the association of rs7038903 with septic shock and 28 days mortality adjusting for clinical covariates. Patients with Callele of rs7038903 were at increasedrisk for development of septic shock (OR=2.44 95%CI 0.96-6.21; P=0.060) as compared to patients with T allele. This increased risk remained after adjustment for age, gender, ancestry, surgical methods and qSOFA scores (OR=2.58; 95%CI 0.98-6.81; P=0.056). We also observed that patients with rs7038903 had a significantly increased hazard of death over the 28 days observation independent of age, gender, ancestry, surgical method and qSOFA score in the Cox regression model (adjusted HR 2.96; 95%CI 1.10-7.94; *p* = 0.031). Moreover, the association between rs7038903 and increased hazard for death remained significant after further adjusting for cytokines level. Interestingly, significant differences were seen in plasma IL6 in individuals with or without rs7038903 C allele (28pg/ml (IQR 12-86) vs 90pg/ml (IQR 49-155); (*p* = 0.022) in patients with severe sepsis in the Malay ethnic group.

## DISCUSSION

The incidence of septic shock and mortality were similar regardless of race. However, there were notable racial disparities in association of cytokines level and septic shock and all-cause mortality in Asian population. Further studies in larger Asian populations are warranted to validate our findings. Although age in general is a risk factor for poor outcomes in sepsis, significant difference in age was observed among various ethnicities. The proportion of septic young patients with poor outcomes (less than 65 years old) was highest among Malays.

SVEP1 is similar to the selectin family of adhesion proteins and was previously proven to be a cell surface protein involved in cell adhesion processes. A recent study demonstrates that SVEP1 is a ligand for integrin a9ß1 and mediates cell adhesion protein is composed of 3,571 amino acids having multiple structural domains including Sushi domain, von Willibrand factor type A domain, epidermal growth factor domain, hyaline repeat domain, and pentraxin domains SVEP1 expression detected in the tissues of heart, lung, skeletal tissue, placenta, stomach, intestine, stromal osteogenic tissues (periosteum and bone), and bone marrow mesenchymal stromal cells, endothelial cells, and breast carcinoma cells^10^. Sushi domains are short consensus repeats belonging to the family of complement control proteins (CCPs). CCP protein modules are of approximately 60 amino acids and have protein-protein interaction domains with many other proteins that regulate blood coagulation andcomplement systems. Our studyshowsa promising polymorphism in SVEP1 gene (rs7038903) which is associated with sepsis shock and 28 days mortality, independent of age, gender, and method of diagnosis and SOFA score. Collectively, while our findings so far have shown the additional value or measuring cytokines and genetic markers in sepsis outcomes in the local population, further large scare studies are neededina heterogeneous septicpopulation with a rigorous analysis to know the significance of our findings.

## CONCLUSION

To our best knowledge, our study is the first to perform genotype profiling in severe sepsis population in Singapore and prospectively determine the association with sepsis shock and mortality. Furthermore, we used multiple mutation prediction analysis tools used for function prediction to identify the variant with deleterious impact. Several limitations of our study should be acknowledged. Firstly, we did not correct for multiple genotyping comparisons given our modest sample size. Furthermore, a replication in other sepsis population would provide more confidence to our data. While we used multiple analysis tool to predict functional impact of rs7038903, further function studies using knock out models would verify the causality.
